# Phenotypic T Cell Exhaustion in a Murine Model of Bacterial Infection in the Setting of Pre-Existing Malignancy

**DOI:** 10.1371/journal.pone.0093523

**Published:** 2014-05-05

**Authors:** Rohit Mittal, Maylene Wagener, Elise R. Breed, Zhe Liang, Benyam P. Yoseph, Eileen M. Burd, Alton B. Farris, Craig M. Coopersmith, Mandy L. Ford

**Affiliations:** 1 Department of Surgery and Emory Center for Critical Care, Emory University School of Medicine, Atlanta, Georgia, United States of America; 2 Department of Surgery and Emory Transplant Center, Emory University School of Medicine, Atlanta, Georgia, United States of America; 3 Department of Pathology and Laboratory Medicine, Emory University School of Medicine, Atlanta, Georgia, United States of America; University of Cincinnati, United States of America

## Abstract

While much of cancer immunology research has focused on anti-tumor immunity both systemically and within the tumor microenvironment, little is known about the impact of pre-existing malignancy on pathogen-specific immune responses. Here, we sought to characterize the antigen-specific CD8^+^ T cell response following a bacterial infection in the setting of pre-existing pancreatic adenocarcinoma. Mice with established subcutaneous pancreatic adenocarcinomas were infected with *Listeria monocytogenes*, and antigen-specific CD8^+^ T cell responses were compared to those in control mice without cancer. While the kinetics and magnitude of antigen-specific CD8^+^ T cell expansion and accumulation was comparable between the cancer and non-cancer groups, bacterial antigen-specific CD8^+^ T cells and total CD4^+^ and CD8^+^ T cells in cancer mice exhibited increased expression of the coinhibitory receptors BTLA, PD-1, and 2B4. Furthermore, increased inhibitory receptor expression was associated with reduced IFN-γ and increased IL-2 production by bacterial antigen-specific CD8^+^ T cells in the cancer group. Taken together, these data suggest that cancer's immune suppressive effects are not limited to the tumor microenvironment, but that pre-existing malignancy induces phenotypic exhaustion in T cells by increasing expression of coinhibitory receptors and may impair pathogen-specific CD8^+^ T cell functionality and differentiation.

## Introduction

Cancer cells have multiple immune modulatory effects by which they are able to evade immune responses [1]–[5]. Various mechanisms of tumor escape have been described in multiple tumor models including breast cancers, skin cancers, pancreatic adenocarcinomas, ovarian cancers, etc. [1], [3], [6]. These include alterations in tumor antigens, the loss of which is seen in patients with recurrent metastatic melanoma [7]. Also, tumor cells are implicated in causing alterations within their microenvironment resulting in downregulation of immune responses, particularly by upregulation of local regulatory T cells (T_regs_), myeloid derived suppressor cells (MDSCs), and tumor-induced apoptosis of tumor antigen-specific cytotoxic T cells [8] leading to impaired tumor specific immunity.

Tumor associated immune modulation is associated with upregulation of inhibitory receptors, such as B and T lymphocyte attenuator (BTLA), PD-1, and 2B4, on T cells [9], leading to T cell exhaustion, during which lymphocytes progressively lose effector function and upregulate expression of inhibitory receptors [9], [10]. PD-1 blockade in mice results in reduction in ovarian tumor size, and increased tumor-specific T cell immune responses [11]. Increased BTLA and PD-1 expression is noted on tumor-specific CD8^+^ T cells in patients with metastatic melanoma and anti-BTLA and anti-PD-1 immunotherapy has shown to improve tumor-specific CD8^+^ T cell immune responses with increased frequencies of IFN-γ, TNF, and IL-2 cytokine producing CD8^+^ T cells [12]. Additionally, pharmacologic blockade of the coinhibitory CTLA-4 and PD-1 pathways are current therapeutic targets in patients with advanced melanoma [13]. Targeting these pathways for the reversal of T cell exhaustion and augmentation of tumor-specific immune responses is the focus of multiple studies in various malignancies [14]–[16].

While the ability of tumors to induce a localized immune suppressive environment [17] is well-appreciated, the impact of malignancy on systemic immune responses has not been extensively explored. However, it is known that cancer patients are predisposed to developing opportunistic infections like those observed in immunocompromised hosts (e.g. pregnancy, pharmacologic immunosuppression) [18]. *L. monocytogenes* is a foodborne bacterium that preferentially infects such patients. Among nonpregnant patients, pre-existing malignancy is the second most common co-morbidity associated with the development of human listeriosis [18], [19]. Epidemiological studies looking at septic patients reveal that septic patients with malignancy have higher mortality than patients without malignancy [20]. Furthermore, recent data in septic mice revealed that mice with a pre-existing malignancy have a 24% greater mortality following a septic insult compared to wild type and that this is mediated by the immune system [21], [22]. This intriguing result suggested that the presence of cancer may alter the systemic immune response to a microbial insult, and prompted us to examine the impact of pre-existing malignancy on microbe-specific T cell responses following bacterial infection.

## Methods

### Ethics statement

All experiments were performed in accordance with the National Institutes of Health Guidelines for the Use of Laboratory Animals and were approved by the Institutional Animal Care and Use Committee at Emory University School of Medicine (Protocol 2001875-082815BN).

### Mice

Adult male 6-week old C57BL/6 were obtained from The Jackson Lab (Bar Harbor, ME). After allowing the mice to acclimate for one week, they were randomized to cancer and control groups. TCR Transgenic OT-I mice were purchased from Taconic and bred onto a Thy1.1^+^ background. Animals were sacrificed at predetermined endpoints using asphyxiation by CO2. All animals were housed in the biosafety facility and had access to chow and water. Following infection with *Listeria monocytogenes*, mice were examined daily to determine if any of the animals were moribund. The following criteria was used to identify moribund animals: clinical or behavioral signs unresponsive to appropriate intervention persisting for 24 hours including significant inactivity, labored breathing, sunken eyes, hunched posture, piloerection/matted fur, one or more unresolving skin ulcers, and abnormal vocalization when handled. Throughout this study, no animals died due to infection and no moribund animals were noted.

### Cancer model

A syngeneic mouse pancreatic adenocarcinoma cell line Pan02 (a generous gift from Dr. David Linehan, Washington University, St. Louis) was used to induce cancer [23], [24]. Cells were maintained in 1640 RPMI culture medium supplemented with 10% fetal bovine serum (FBS), 1% glutamine, penicillin/streptomycin, and 4-(2-hydroxyethyl)-1-piperazineethanesulfonic acid (HEPES) [21], [22]. Mice stratified to the cancer group underwent a subcutaneous injection of 250,000 cells in 0.1 mL of PBS in the right inner thigh. Mice were then housed in the animal facility for a 3-week period during which palpable non-metastatic tumors developed. Control mice were unmanipulated.

### T cell adoptive transfer

Spleens were collected from TCR transgenic OT-I mice bred onto a Thy1.1^+^ background. Single cell suspensions were prepared and counted, and splenocytes were stained with anti-Vα2, anti-Vβ5, and anti-CD8 (Pharmingen, San Diego, CA) to determine the frequency of OT-I^+^ T cells within the suspension. All mice received a single IV injection of 10^4^ OT-I^+^ T cells along with syngeneic carrier splenocytes 3 weeks after tumor cell injection and 24 h prior to Listeria infection.

### Listeria monocytogenes-OVA infection model

To model infection, we used the intracellular bacterium *Listeria monocytogenes*, which is cleared by day 7 in wild-type B6 mice [25]. Specifically, we used a *Listeria monocytogenes* strain with OVA insert (LM-OVA) with streptomycin resistance [26], which was incubated in 5 mL of brain-heart infusion broth (Teknova) supplemented with 50 mg/mL streptomycin at 37°C overnight. 24 hours post-adoptive transfer, all mice were infected with 10^4^ CFU LM-OVA suspended in 0.5 mL sterile PBS via IP injection.

### Specimen collection and flow cytometric analysis for cell frequency and intracellular cytokine staining

Groups of mice were sacrificed at the following time points: uninfected (day 0) and post-infection (days 5 and 14). At the indicated time points, spleens were collected from all animals and single cell suspensions were prepared. Cells were stained with anti-CD4-PO (Invitrogen), anti-CD44-FITC and anti-Thy1.1-PerCP (all from BD Pharmingen), anti-CD8-PB, anti-PD-1-FITC, anti-BTLA-PE, and anti-2B4-allophycocyanin (all from eBioscience).

For intracellular cytokine staining, single-cell suspensions of splenocytes were plated in a 96-well plate (1×10^6^ cells per well) in culture medium containing RPMI 1640 containing 10% FBS (Mediatech, Herndon, VA), 2 mM L-glutamine, 0.01 M HEPES buffer, 100 mg/ml gentamicin (Mediatech), and 5×10^−5^ M 2-mercaptoethanol (Sigma-Aldrich, St. Louis, MO). Cells were incubated for 4 h in 10 nM OVA_257-264_ (SIINFEKL; Emory University Microchemial Core Facility) and 10 mg/ml brefeldin A (Pharmingen). Following incubation, cells were stained with anti-CD4-PO (Invitrogen), anti-Thy1.1-PerCP (BD Pharmingen), and anti-CD8-PB (eBioscience) and processed using an intracellular staining kit (BD Biosciences) and stained with anti-IFN-γ-Alexa 700 and anti-IL-2-FITC (BD Biosciences). All samples were run on a LSRII flow cytometer (BD Biosciences), and data was analyzed using FlowJo 9.5 Software (Tree Star, San Carlos, CA).

### Statistical analysis

Statistical analyses were conducted using GraphPad Prism 5.0 software (San Diego, CA) and presented as mean ± SEM. Two-way comparisons were performed using the Mann-Whitney test or Spearman correlation coefficient and a p value of <0.05 was considered to be statistically significant.

## Results

### In the setting of pre-existing malignancy, acute systemic bacterial infection does not alter antigen-specific T cell expansion

**Figure 1:**
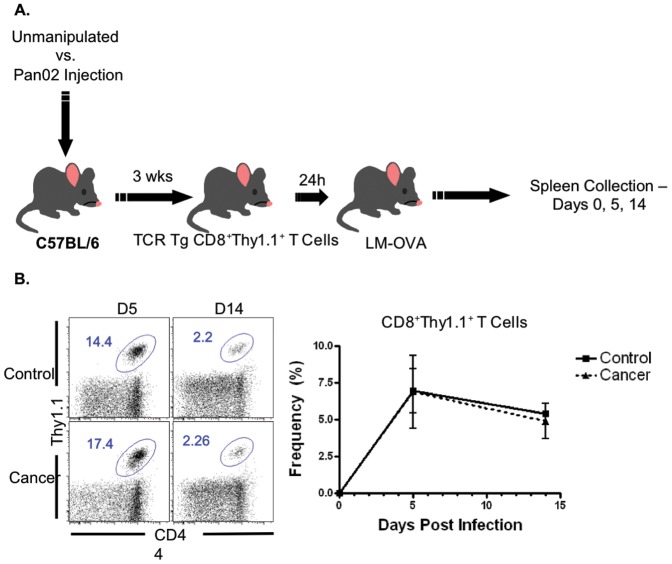
Experimental design and bacterial antigen-specific immune responses. A) C57BL/6 mice were randomized to control vs. cancer groups. The cancer group received a subcutaneous injection of Pan02 cells. Following 3 weeks, all mice were given an IV injection of transgenic (Tg) T cell receptor (TCR) CD8^+^Thy1.1^+^ T cells. After 24 hours, uninfected mice were sacrificed for the day 0 time point, and all other mice were given an intraperitoneal injection of LM-OVA and underwent spleen collections at days 5 and 14 post-infection. B) At days 5 or 14 following infection, there were no significant differences in antigen-specific CD8+T cells expansion between the control and cancer groups. The mean frequencies in control vs. cancer were 6.97±1.49% vs. 6.89±2.49% at day 5, respectively and 5.38±0.73% vs. 4.90±1.19% at day 14, respectively; n = 8–10 at all time points.

We sought to create a model to quantify and characterize pathogen-specific CD8^+^ T cell responses in the setting of pre-existing malignancy as compared to cancer-free mice. In order to characterize the antigen-specific host response, all mice received an IV adoptive transfer of TCR transgenic T cells specific for a class I-restricted epitope derived from chicken ovalbumin (OT-I) [27]. These T cells were congenically marked via expression of Thy1.1. Mice were then infected with a recombinant *Listeria monocytogenes* engineered to express the OVA-derived epitope recognized by OT-I T cells [26]. Infection was introduced via an IP injection 24 hours later. Thus, we were able to characterize the antigen-specific CD8^+^Thy1.1^+^ T cell responses at days 0, 5, and 14 post-infection (figure 1a).

We assessed the frequency of OVA-specific CD8^+^Thy1.1^+^ T cells responding within the CD8^+^ T cell compartment. As expected, there was no detectable antigen-specific CD8^+^ T cell response at day 0. At days 5 and 14 following infection with LM-OVA, no statistically significant differences were noted in the antigen-specific CD8^+^ T cell response between the cancer and control groups (figure 1b). In addition, frequencies of total endogenous CD4^+^ and CD8^+^ T cell compartments were comparable between the two groups (Figure S1).

### Lymphocyte activation following infection is unchanged by the presence of malignancy

**Figure 2:**
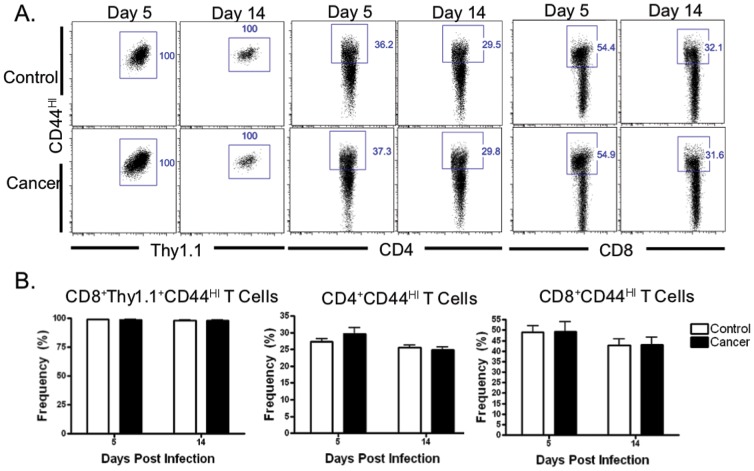
Effect of cancer and LM-OVA infection on T cell activation. No differences were noted in the mean frequency of the activated CD8^+^Thy1.1^+^ T cell response at day 5 (98.89±0.14 vs. 98.81±0.19) and 14 (98.18 ±0.21 vs. 98.32±0.35) in the control vs. cancer groups, respectively. Similarly, activated CD4^+^ T cell frequencies were not different at day 5 (27.37±0.77 vs. 29.67±1.95) or day 14 (25.50±0.84 vs. 24.84±0.85). Also, the frequency of CD8^+^ T cell activation was not different at days 5 (48.97±3.12 vs. 49.18±4.90) and 14 (42.62±2.98 vs. 43.01±3.44) in control vs. cancer, respectively; p = ns and n = 8–10.

Given the similarities in the kinetics and magnitude of the antigen-specific CD8^+^ response between control and cancer mice, we endeavored to further characterize the degree of T cell activation and differentiation in the setting of bacterial infection and pre-existing malignancy. CD44 is critically involved in the processes of effector and memory T cell migration, adhesion, and activation [28]. In our model, T cell activation was measured by assessing frequency of CD44^HI^ cells within CD4^+^, CD8^+^, and CD8^+^Thy1.1^+^ T cells at day 5 (peak of the antigen-specific CD8^+^ T cell response) and day 14 following resolution of infection. No differences were noted in CD44 activation phenotype within the CD8^+^Thy1.1^+^ compartment between the cancer and control groups at either day 5 or 14. Similarly, frequencies of endogenous effector or memory CD4^+^ and CD8^+^ T cell populations were not different at any of the time points (figure 2).

### Pathogen-specific and endogenous T cells demonstrate phenotypic exhaustion in cancer mice following bacterial infection

The expression of the coinhibitory markers 2B4, BTLA, and PD-1 are known to modify CD8^+^ T cell survival and effector function in models of both infection and tumor immunity [9]. Thus, we assessed coinhibitory receptor profiles on Listeria-specific CD8^+^ T cells in control vs. cancer animals.

**Figure 3:**
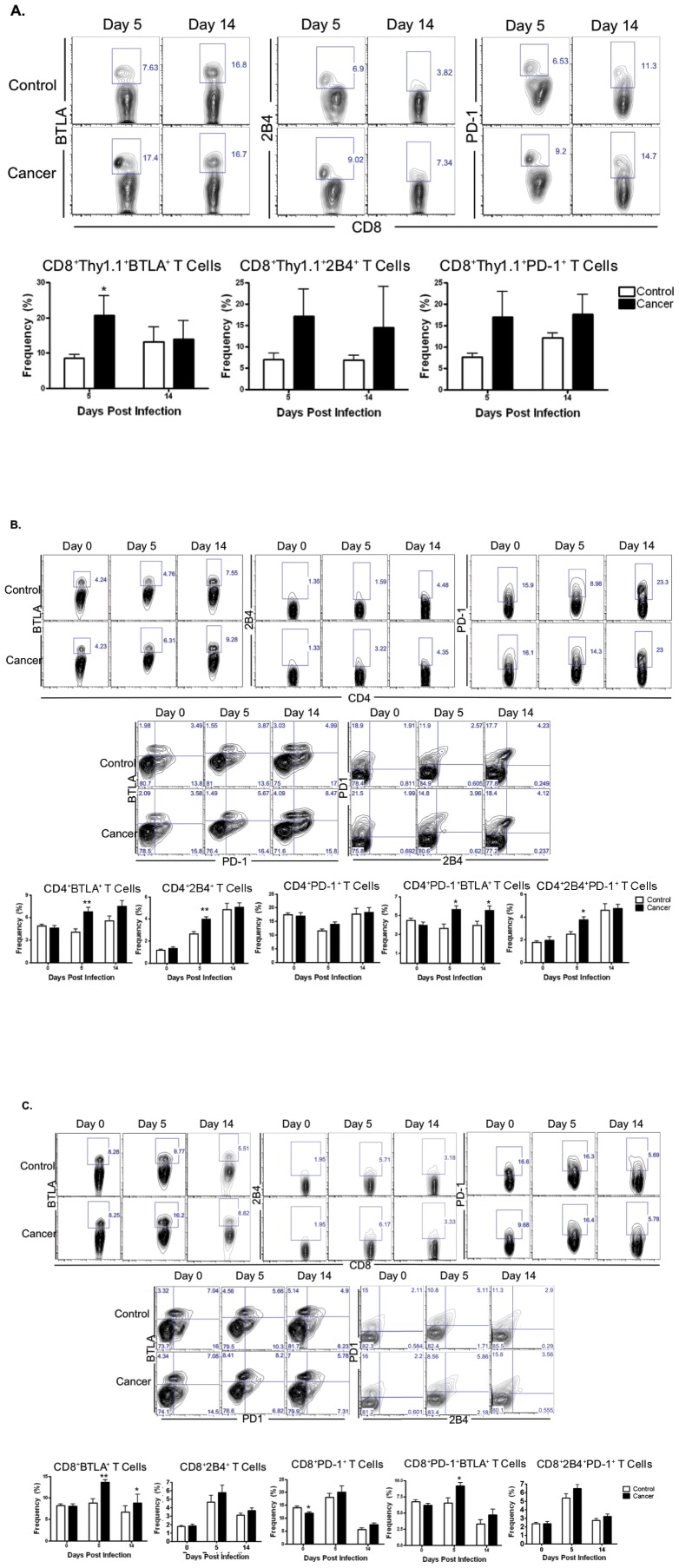
Effect of cancer and LM-OVA infection on coinhibitory receptor expression. A) Data presented is following selection of the CD8^+^Thy1.1^+^ T cell population. CD8^+^Thy1.1^+^ T cells in cancer group had increased mean frequencies of BTLA^+^ T cells. They were 8.58±1.07% in control vs. 20.72±5.58% in cancer mice following infection (*p<0.05; n = 8–10) at day 5. Mean frequencies of 2B4 (n = 4–5) and PD-1 (n = 8–10) had a trend toward increased expression in the cancer group, but were not statistically significant. B) In the absence of infection (day 0), the control and cancer groups did not have any significant differences in the expression or co-expression of 2B4, BTLA, and PD-1 on CD4^+^ T cells. At day 5, however, CD4^+^ T cells in the cancer group expressed a higher frequency of BTLA (4.03±0.46 vs. 6.77±0.59) and 2B4 (2.67±0.22 vs. 3.95±0.25). The inhibitory profile was further altered when looking at co-expression. The cancer group had higher expression of PD-1^+^BTLA^+^ (3.76±0.47 vs. 5.65±0.42) and 2B4^+^PD-1^+^ (2.50±0.23 vs. 3.76±0.26) CD4^+^ T cells. Following resolution of infection at day 14, the expression of 2B4 (n = 4–5), 2B4^+^PD-1^+^ (n = 4–5), or PD-1 was no longer different in both groups, however, the frequency of expression CD4^+^ PD-1^+^BTLA^+^ T cells remained elevated in the cancer group (3.99±0.44 vs. 5.56±0.48). **p<0.01; *p<0.05; n = 8–10 at days 0, 5, and 14, unless otherwise stated. C) At baseline (day 0), cancer mice had reduced expression of CD8^+^PD-1^+^ T cells compared to control mice (14.03±0.61 in control vs. 11.91±0.47 in cancer). However, expression of 2B4, BTLA, 2B4^+^PD-1^+^ or PD-1^+^BTLA^+^ was unchanged prior to infection. At day 5 following infection, cancer mice had increased frequencies of expression of CD8^+^BTLA^+^ (8.78±0.95 vs. 13.66±0.64) and CD8^+^PD-1^+^BTLA^+^ (6.55±0.83 vs. 9.22±0.47) T cells. Following resolution of infection at day 14, only the mean frequency of BTLA expression in the cancer group remained elevated (6.72±1.40 vs. 8.76±2.03); **p<0.01; *p<0.05; n = 8–10.

At day 5 post-infection, CD8^+^Thy1.1^+^ T cells isolated from the cancer group had statistically significant higher frequencies BTLA^+^ cells as compared to the control group. The frequency of 2B4 and PD-1 expressors within the CD8^+^ Thy1.1^+^ populations in the cancer group trended higher as well, however, this difference was not statistically significant (figure 3a).

We also assessed coinhibitory receptor expression levels on endogenous CD4 and CD8 T cell (figures 3b and 3c, respectively). In order to determine if any phenotypic changes were due to the presence of a malignancy, we first determined the baseline inhibitory profile at day 0 prior to LM-OVA infection. While we failed to observe significant changes in coinhibitor expression on endogenous CD4^+^ T cells in control vs. cancer animals at baseline, endogenous CD8^+^ T cells in the control group exhibited increased expression of PD-1 (Figure 3c). At day 5 post-infection, endogenous CD4^+^ T cells in the cancer group had an inhibitory receptor profile consistent with phenotypic exhaustion with increased expression of BTLA and 2B4. Additionally, cancer mice exhibited increased co-expression of CD4^+^PD-1^+^BTLA^+^ and CD4^+^2B4^+^PD-1^+^ T cells at day 5 (Figure 3b). Similarly, the endogenous CD8^+^ T cell population in the cancer group also exhibited increased expression of CD8^+^BTLA^+^ and CD8^+^PD-1^+^BTLA^+^ T cells at day 5 post-infection (figure 3c). While all were able to survive the infection, at day 14, endogenous CD4^+^ and CD8^+^ T cell compartments in cancer mice continued to contain higher frequencies of CD4^+^PD-1^+^BTLA^+^ and CD8^+^BTLA^+^ T cells.

### Increased inhibitory receptor expression on antigen-specific CD8^+^ T cells is associated with altered cytokine production

**Figure 4:**
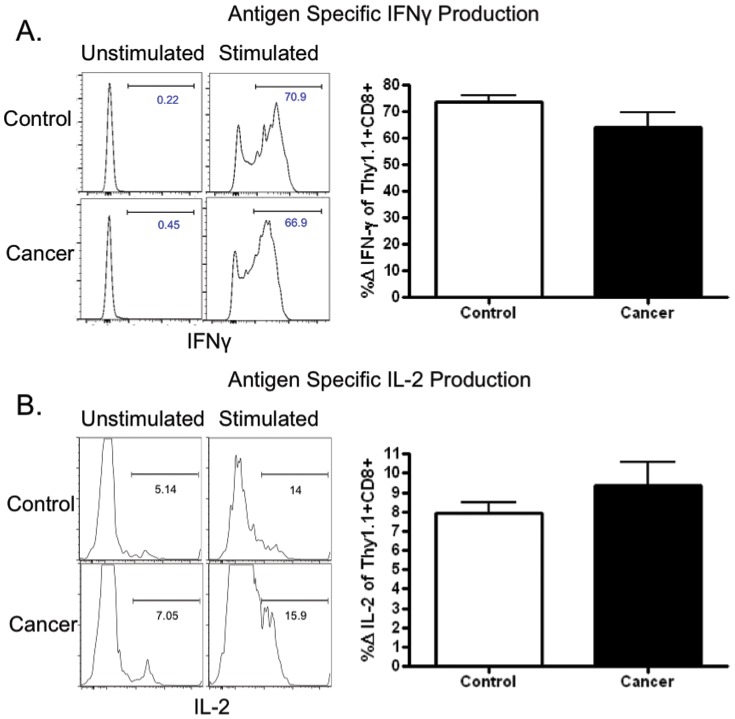
Antigen-specific T cell cytokine production in control and cancer mice following intracellular cytokine stimulation. CD8^+^Thy1.1^+^ T cells in the control and cancer groups were stimulated with SIINFEKL peptide. No statistically significant differences were noted in intracellular production of IFN-γ or IL-2; n = 8–10.

**Figure 5:**
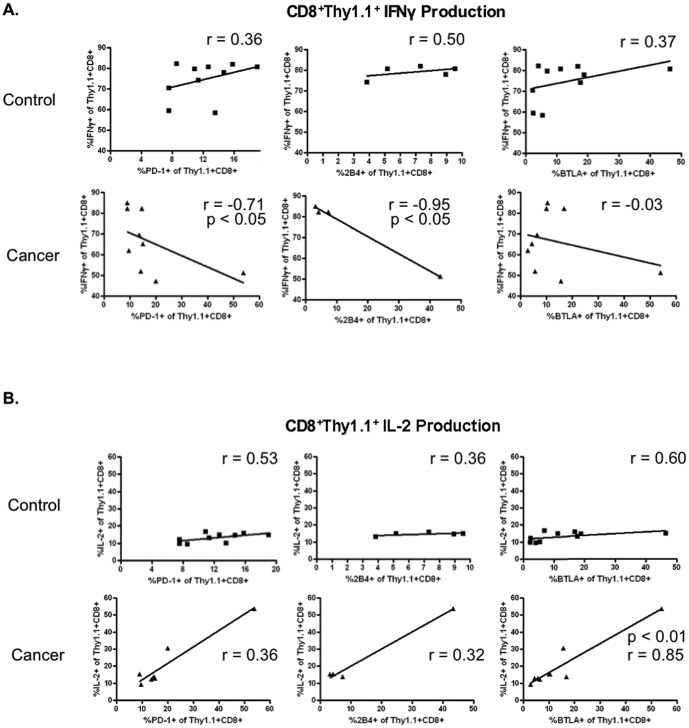
Correlation of coinhibitory receptor expression to antigen-specific cytokine production. A) The frequency of PD-1, 2B4, and BTLA expression was compared to the frequency of cells producing IFN-γ. Increased inhibitory receptor expression is correlated with reduced cytokine production. n = 8–10 (PD-1 and BTLA) and 4–5 (2B4). B) The frequency of PD-1, 2B4, and BTLA expression was compared to the frequency of cells producing IL-2. Increased inhibitory receptor expression is correlated with increased cytokine production; n = 8–10 (PD-1 and BTLA) and 4–5 (2B4).

At day 14 post-infection, the frequencies of IFN-γ and IL-2 producing cells in the antigen-specific CD8^+^Thy1.1^+^ T cell compartment were not statistically different in cancer mice vs. non-cancer controls (figure 4). However, CD8+ Thy1.1+ antigen-specific T cells exhibited a trend toward reduced IFN-γ and higher IL-2 production; therefore, we sought to determine if this trend correlated with the degree of coinhibitory molecule expression in this compartment. We plotted frequencies of PD-1^+^, 2B4^+^, and BTLA^+^ cells within the CD8^+^ Thy1.1^+^ population of a given animal against the frequencies of IFN-γ^+^ or IL-2^+^ CD8^+^ Thy1.1^+^ T cells within the same animal. We observed that increased PD-1 and 2B4 expression on antigen-specific CD8^+^ T cells correlated with reduced IFN-γ production, and that increased BTLA expression demonstrated a statistically significant association with increased IL-2 production in the antigen-specific T cells (Figures 5a and 5b). These associations were only observed in cancer mice and not in non-cancer controls.

## Discussion

Our study revealed differences in the expression of coinhibitory receptors on pathogen-specific CD8^+^ cells responding to infection in the context of an established tumor. Specifically, in the cancer group, bacterial antigen-specific CD8^+^ T cells showed increased BTLA expression; PD-1 and 2B4 expression trended higher in the cancer group but was not significant. Importantly, however, the expression of these coinhibitory molecules correlated with reduced T cell inflammatory effector function as measured by IFN-γ production. Expression of BTLA also correlated with increased IL-2 production, which is potentially suggestive of a less terminally differentiated effector state. Thus, the data presented here provide evidence that tumor-associated immune suppression is not limited to the microenvironment, but that the presence of malignancy may induce an exhausted phenotype in pathogen-specific T cells following bacterial infection.

Coinhibitory receptors are known to play important roles in regulating antigen-specific T cell responses in models of anti-viral immunity, autoimmunity, and transplantation. During malignancy, upregulation or persistent expression of inhibitory markers, especially PD-1 and BTLA, is a documented means of immune-evasion by malignant cells [29], [30]. For example, in patients with melanoma (presumably a setting of immune incompetence), BTLA expression remains high in tumor antigen-specific CD8^+^ T cells [30], [31]. Furthermore, Riches et al. noted chronic lymphocytic leukemia specific CD8^+^ T cells exhibited increased PD-1 expression and an exhausted phenotype, despite no functional difference in cytokine production [32], while Amarnath et al. showed conversion of CD4^+^ T cells to T regulatory cells following stimulation of the PD-1 receptor by its ligand PD-L1 [33]. PD1/PD-L1 blockade has also been shown to restore functionality to exhausted tumor-specific CD8^+^ T cells in vitro [12]. Finally, tumor microenvironments have increased PD-L1/PD-1 interactions taking place following lymphocyte infiltration [34] with blockade of these interactions enhancing antitumor activity [35], [36]. Clinical trials of PD-1 blockade in metastatic melanoma are underway. Given that we see PD-1 expression induced by bacterial infection in cancer mice, our data suggest that targeting this pathway may improve outcomes in the management of infections in cancer patients.

Our cancer model also revealed increased expression of 2B4, a CD2 Ig superfamily member, following infection. These results are consistent with previously reports in models of both cancer and chronic viral infection in which high 2B4 expression was noted to impair antigen-specific CD4^+^ responses to *Mycobacterium tuberculosis* during the active and latent phases of infection [37]. With regard to CD8^+^ T cells, co-expression of both 2B4 and PD-1 has been purported to impact proliferative capacity and differentiation during acute and chronic HCV infection [38], [39]. In a model of melanoma, tumor-specific CD4^+^ effector T cells demonstrated exhaustion and increased 2B4 and PD-1 expression [40]. Thus, while these findings pose important implications in the treatment of melanoma, our data now suggest that immune modulatory therapies targeting these receptors may also play a role in the management of bacterial infections and restoring immune responses in cancer patients.

Our data also reveal that lower IFN-γ production and increased IL-2 production correlated with increased coinhibitory molecule expression in the cancer model. These results are consistent with the fact that less IL-2 production and more IFN-γ production are associated with increased terminal T cell differentiation [41]. Increased terminal differentiation may occur in the presence of increasing amount or duration of antigenic stimulus [42]. Thus, we can speculate that tumors might impact T cell differentiation by reducing the expression of peptide:MHC complexes on the surface of antigen presenting cells. Alternatively, terminal T cell differentiation can also be mediated by the inflammatory stimulus IL-12 (signal 3) [43]–[45]. Thus, we might also hypothesize that the presence of cancer may inhibit APC activation and differentiation to reduce the provision of signal 3, resulting in blunted T cell differentiation in these animals.

Our study sheds light on the way in which the adaptive immune response to acute bacterial infection is modified in a cancer host. Previous data published by our lab suggest that lymphocytes also play an important role in modulating the increased mortality seen in septic cancer mice [21], [22]. Upregulation of BTLA on lymphocytes and PD-1 on macrophages is noted to contribute to increased mortality seen in sepsis [46], [47]. Given our findings demonstrating increased expression of coinhibitory molecules on bacteria-specific CD8^+^ T cells in the setting of pre-existing malignancy, we speculate that this upregulation of costimulatory molecules may underlie the increased mortality observed in septic cancer animals, where the bacterial burden may make T cells more likely to undergo exhaustion. Future investigation to interrogate the functional role of coinhibitory receptors in the setting of cancer-induced sepsis mortality is warranted.

Our data revealed differences in the constellation of coinhibitory molecules co-expressed on antigen-specific CD8^+^ T cells vs. endogenous polyclonal CD4^+^ or CD8^+^ T cells in Listeria-infected animals in the setting of cancer. While OVA-specific TCR transgenic T cells are a monoclonal population responding to a known epitope on our *Listeria* strain, the endogenous polyclonal CD4^+^ and CD8^+^ T cell populations are likely specific for numerous other epitopes with varying affinities for pMHC, densities of expression on the cell surface, and distribution on distinct APC subsets. We speculate that these differences may contribute to the discrete coinhibitory receptor profiles observed on endogenous polyclonal CD4^+^ and CD8^+^ and antigen-specific CD8^+^ T cells. Understanding the effect of cancer on distinct T cell subsets responding to bacterial challenge remains an important goal.

In this study, we use a model of malignancy to study bacterial antigen-specific CD8^+^ T cell immune responses. Future assessment of antigen-specific CD4^+^ T cell responses could further enhance our understanding of the impact of pre-existing malignancy on protective immunity. In addition, our observations of phenotypic T cell exhaustion were made in the setting of pancreatic adenocarcinoma, and it is not yet clear whether similar results would be obtained in alternative models of malignancy. For example, LM-OVA infection is non-lethal in our model and cleared by all mice with no statistical difference in bacterial burden between cancer and non- cancer mice (Figure S2), and future studies in the setting of higher mortality models of pre-existing malignancy could provide a more complete understanding of how the immune system responds to microbial antigens during the state of cancer-induced immune dysregulation.

In summary, our findings suggest that immunosuppressive effects of cancer are not a localized phenomenon but rather may contribute to a state of generalized immune suppression leading to phenotypic exhaustion and impaired T cell differentiation.

## Supporting Information

Figure S1 **Effect of cancer and LM-OVA on CD4^+^ and CD8^+^ T cell populations.** No differences were noted in the frequency of (A) CD4+ T cells in splenocytes at days 0 (8.45±0.34 vs. 7.92±0.60), 5 (8.99±1.17 vs. 8.67±0.94), and 14 (10.59±0.53 vs. 10.88±0.41) in control vs. cancer mice, respectively. B) Similarly, frequencies of CD8+T cells were not statistically significant at at day 0(5.83±0.16 vs. 5.55±0.40), 5 (8.76±0.74 vs. 8.81±0.40), and 14 (8.67±0.50 vs. 8.64±0.48) in control vs. cancer mice, respectively; n = 8–10.
(EPS)

Figure S2 **Results of bacterial tissue culture following LM-OVA infection.** No differences were noted in bacterial colony forming units (CFU/mg) in control vs. cancer animals at day 2 (25.44±24.64 vs. 1.86±1.37; n = 4–5) and both groups cleared infection by day 5 (0.02±0.02 in control vs. 0.02±0.02 in cancer; n = 9–10) post-infection with LM-OVA; p = ns for both time points.
(EPS)

Methods S1 **Supporting methods for culture of** ***Listeria monocytogenes*** **from splenocytes.**
(DOCX)
