# Supplementary material for: Phenotypic T Cell Exhaustion in a Murine Model of Bacterial Infection in the Setting of Pre-Existing Malignancy
Source: PLoS One. 2014 May 5;9(5):e93523. doi: 10.1371/journal.pone.0093523 (PMC4010417; doi:10.1371/journal.pone.0093523)
Supplement: Methods S1 — Supporting methods for culture of Listeria monocytogenes from splenocytes. (DOCX) [file pone.0093523.s003.docx]

Supporting Information:

Bacterial Culture Methods:

*Spleen culture for Listeria monocytogenes colonies*

Spleens samples were collected from non-cancer and cancer mice infected with LM-OVA at days 2 and 5 post-infection and weighed to the nearest 0.1mg using sterile technique. Each tissue sample was suspended in 120 µl of sterile 0.85% saline and homogenized using a small disposable sterile Kendall tissue grinder (Covidien, Mansfield, MA).  Serial 10-fold dilutions were prepared in sterile 0.85% saline.  A 100 µl aliquot of undiluted sample and each dilution from 10^-1^ to 10^-3^ was plated on blood agar plates (Remel, Lenexa, KS) and incubated at 35°C in a 5% CO2 atmosphere for 24 hours. Colony counts were obtained from plates containing fewer than 300 colonies.  The number of colony forming units per milligram of tissue (CFU/mg) was determined by multiplying the number of colonies (adjusted for the volume of diluent used for tissue homogenization) by the reciprocal of the dilution counted and divided by the weight of the sample in milligrams.
